# A Literature Review on the Anesthetic Management of Pulmonary Arterial Hypertension in Non-cardiothoracic Surgery

**DOI:** 10.7759/cureus.39356

**Published:** 2023-05-22

**Authors:** Joseph M Hendrix

**Affiliations:** 1 Anesthesiology, Academic Medical Center, Irving, USA

**Keywords:** perioperative management, perioperative evaluation, anesthesiology, non-cardiothoracic surgery, pulmonary hypertension

## Abstract

Pulmonary hypertension (PH) is characterized by narrowing small pulmonary arteries, escalating pulmonary vascular resistance, and affecting the entire cardiovascular system. Pulmonary arterial hypertension (PAH) represents a subgroup of PH and typically affects one in 20,000 individuals. When treating individuals with PAH for non-cardiothoracic surgery, anesthetic management strategies should be tailored to the individual's specific needs. This literature review assessed the anesthetic management of PAH in non-cardiothoracic surgery. Electronic databases such as PubMed, ScienceDirect, Ovid Medical Literature Analysis and Retrieval System Online (MEDLINE), Cochrane, and Google Scholar were searched using relevant keywords related to PAH, noncardiac surgery, and anesthesia. Reference lists and author names were also investigated. The articles that met the inclusion criteria provided evidence suggesting that preoperative assessment should be comprehensive, hemodynamic goals should be established, anesthesia providers should be familiar with the underlying pathology, and cross-consultations between surgeons and anesthesia providers are essential for achieving satisfactory outcomes. Furthermore, patient care should extend beyond the surgical procedure into postoperative recovery in the post-anesthesia care unit (PACU) or intensive care unit (ICU) setting. In conclusion, it is vital for anesthetic management strategies to accommodate the unique needs of PAH patients to optimize their safety during perioperative care.

## Introduction and background

Pulmonary hypertension (PH) is when the mean pulmonary arterial pressure (mPAP) is higher than normal, exceeding 20 mmHg at rest. This is often detected through right heart catheterization (RHC) but can also be estimated using the formula PAP = LAP + (CO × PVR)/80, wherein LAP stands for left atrial pressure, CO denotes cardiac output, and PVR stands for pulmonary vascular resistance [1,2].

Under healthy conditions, the pulmonary vasculature functions as a low-resistance/high-flow system. However, in cases of pulmonary arterial pressure (PAH), this balance is disrupted due to the progressive constriction of the small pulmonary arteries, which leads to increased PVR and mPAP [3]. This decreases CO and can eventually result in right heart failure (RHF).

Left atrial pressure may be increased due to left ventricular (LV) failure or valve disease. In addition, elevated CO can arise from a congenital heart disorder, pregnancy, hyperthyroidism, sepsis, liver cirrhosis, anemia, or beriberi [4]. It may also be reduced or normal in either precapillary or postcapillary PH [4]. Heightened PVR can be acute or chronic [5]. Impending increases in acute PVR might be triggered by hypoxia, hypercapnia, acidosis, increased sympathetic tone, pulmonary vasoconstrictors, or pulmonary embolism. Long-lasting elevations of PVR could occur due to parenchymal lung illness, hypoxia without lung tissue damage such as hypoventilation or altitude sickness, pulmonary embolism (PE), or PAH itself. The underlying causes encompass the contraction of pulmonary artery smooth muscle cells resulting in vascular narrowing, the hypertrophy of intimal cells and adventitia that could develop into fibroblasts and progenitor cells resulting in the thickening of vessel walls, and fibrin clots with fibrinogen forming inside the affected vessels [5].

## Review

Epidemiology

PH demonstrates a general prevalence of one in 20,000 individuals [1]. PAH, a subgroup of PH, has an incidence of approximately 15-50 cases per million adults. PH is observed in 10%-15% of patients with scleroderma or systemic sclerosis, 10%-15% of HIV-infected patients, and 6%-16% of those with portal hypertension (end-stage liver disease) [1-3]. Moreover, 10% of congenital heart disease patients have PH, increasing to 30% if the condition is unrepaired [4]. Approximately 90% of severe chronic obstructive pulmonary disease (COPD) patients develop PH, with 5% of these cases being severe [5]. Interstitial lung disease has a prevalence of 8%-32% among PH patients [6]. Obstructive sleep apnea (OSA) patients have a 15%-20% risk of PH, while rheumatoid arthritis and sickle cell anemia patients have a 15% and 20%-40% risk, respectively [1-6]. In moderate to severe PAH, the perioperative mortality rate is 7%-10% in noncardiac surgeries [1]. However, when portopulmonary hypertension with an mPAP of >50 mmHg is present in liver transplantation, the perioperative mortality rate approaches 100% [1-6].

Mortality rates over time depend on disease severity, which is determined by CO, mPAP, PVR, and right atrial pressure (RAP) [1]. In asymptomatic and compensated disease, CO and RAP remain stable, whereas mPAP and its associated PVR begin to increase [1]. In symptomatic or decompensating disease, mPAP and PVR rise, but RAP remains relatively stable, and CO starts to decline [1]. Finally, CO falls sharply in overt right-sided heart failure, mPAP decreases, and PVR and RAP steadily rise [1].

Diagnosis

PH symptoms can be categorized as early or late [1,6-8]. Early indicators may include an early ejection click, an accentuated pulmonary component of the second heart sound, a mid-systolic ejection murmur, a parasternal heave, an S4 gallop, and a prominent jugular "a" wave [1,6-8]. Late indicators may encompass diagnostic pulmonary regurgitation murmur, holosystolic tricuspid regurgitation murmur, jugular venous distention, a prominent "V" wave, an S3 ventricular gallop, pulsatile hepatomegaly, peripheral edema, and ascites [1,6-8]. Furthermore, the early symptoms of PH can include dyspnea on exertion (80% of patients) and exercise intolerance, while late symptoms may involve fatigue, chest pain, edema, syncope, cough, and dyspnea at rest (10%-30% incidence) [1,6,8].

Typical electrocardiogram (ECG) findings in PH patients may include right ventricular hypertrophy (RVH) resulting in right axis deviation (RAD) (+210°), an elevated V1 R wave and QR complex, prominent V5-6 S waves, V1-3 inverted T waves, ST depression (right ventricular {RV} "strain"), and lead II peaked P-waves (right atrial {RA} enlargement) [1]. Additionally, imaging findings of PH may comprise hilar enlargement, prominent pulmonary interstitial markings, attenuated peripheral and large central pulmonary arteries, RV and right atrial (RA) enlargement, left ventricular (LV) enlargement, and a decreased retrosternal space due to RV enlargement on X-ray imaging [1,6,8]. Moreover, computed tomography (CT) can reveal the dilation of the pulmonary artery trunk. At the same time, magnetic resonance imaging (MRI) may demonstrate ventricular septal bowing that compresses the left ventricle [1,6,8].

Right heart catheterization (RHC) remains the gold standard for diagnosing PAH. The diagnostic criteria are met when mPAP is greater than 20 mmHg, and the pulmonary artery occlusion pressure (PAOP) is less than 15 mmHg [1,2]. Echocardiography can suggest severe PH with RV enlargement and hypertrophy, tricuspid regurgitation with a high-velocity regurgitant jet, a mid-systolic "notch" on pulmonary artery Doppler flow tracing, and a leftward shift of the interventricular septum [6,7]. However, echocardiographic pressure measurements obtained via RHC have an inherent error of ±10 mmHg in approximately 50% of PH patients [2,8-10]. In addition, transthoracic echocardiography is only about 80% sensitive and 75% specific for PH [2].

PH can be classified based on severity, function, or etiology [2,3,7,11-20]. The severity of PH is divided into three major diagnostic levels: mild PH with mPAP of 20-40 mmHg, moderate PH with mPAP of 40-55 mmHg, and severe PH with mPAP greater than 55 mmHg [2,3,7,11-20]. The functional assessment of PH is based on four classifications according to the New York Heart Association/World Health Organization (NYHA/WHO), ranging from no limitations of physical activity (NYHA class I) to physical disability with signs of right heart failure or fatigue at rest (NYHA class IV) [2,7,11-18]. PH can also be classified into five categories based on etiology: PAH, PH caused by left heart disease, PH from pulmonary diseases and/or hypoxemia, chronic thromboembolic pulmonary hypertension (CTEPH), and PH with unclear or multifactorial mechanisms [2,3,7,11-20]. PAH can be generally distinguished from other PH groups or categories by the PAOP of less than 15 mmHg, the lack of history of chronic lung or heart disease or dysfunction before a finding of elevated PVR and mPAP, and the lack of objective test results consistent with significant lung or left ventricle pathology or dysfunction before a finding of elevated PVR and mPAP [1-3].

Treatment strategies for PH depend on the underlying disease, and any secondary underlying diseases should be treated first [7,11-18,20-22]. Testing for the inclusion or exclusion of the secondary causes of PH may include angiography, arterial blood gas (ABG) analysis, polysomnography, serology (HIV, antinuclear antibody {ANA}, and liver function tests {LFTs}), thyroid-stimulating hormone (TSH) test, transesophageal echocardiography (TEE), exercise echocardiography, ECG, and RHC according to the Journal of the American College of Cardiology (JACC)/American Heart Association (AHA) pulmonary hypertension diagnostic algorithm [7,11-18,20-22]. Anticoagulation (to an international normalized ratio {INR} of 2.0-2.5), oxygen therapy if saturation is less than 90%, salt/fluid restriction, and patient/family education are recommended for primary PH [7,11-18,20,22]. Digoxin may be indicated when RHF is present, and any exacerbating factors should be circumvented [7,11-18,20,22]. The patients can be stratified as "lower risk" or "higher risk" based on evaluations and responses to therapies offered [7,11-18,20]. Medications for PH treatment include phosphodiesterase-5 (PDE-5) inhibitors, prostanoids, endothelin receptor antagonists, and calcium channel blockers [7,11-18,20].

Preoperative considerations

For patients undergoing non-cardiothoracic surgeries, anesthesia providers should contemplate local, regional, or epidural sedation if positive pressure ventilation (PPV) is unnecessary. Intrathecal anesthetics are relatively contraindicated due to the risk of acute systemic vascular resistance (SVR) shifts, potentially leading to RV ischemia [21-26]. Preoperative assessment should encompass the evaluation of signs and symptoms, brain natriuretic peptide (BNP) levels, ECG, and imaging studies [21,23,27-30]. In PH patients with RV dysfunction or with severe PH, it is appropriate, before the induction of anesthesia, to begin initiating inotropic agents and pulmonary vasodilators, such as nitric oxide (NO), before induction. NO can be routinely administered by endotracheal tubes, supraglottic airway devices such as laryngeal mask airways, and nasal cannula or face masks. Inotropic agents, such as dobutamine or epinephrine, and pulmonary vasodilators, such as milrinone or NO, should be started and titrated to a balanced effect, avoiding excessive hypotension or tachycardia, prior to the induction of anesthesia in patients with known pre-existing PH with RV dysfunction or RHF or severe PH.

During the preoperative period, anesthesia providers must assess patient risk factors to appraise the risks and benefits of surgery. These factors should include medical history (PE, coronary artery disease {CAD}, and chronic kidney disease {CKD}), New York Heart Association/World Health Organization (NYHA/WHO) classification, right axis deviation (RAD) on ECG, right ventricular hypertrophy (RVH) or a right ventricular myocardial performance index of 0.75, and abnormal hemodynamics (elevated mPAP, RV systolic pressure {RVSP}/systolic blood pressure {SBP} ratio of >0.66) [14,20]. Furthermore, operative risk factors in PAH patients encompass emergency surgery, intermediate- or high-risk operations, higher American Society of Anesthesiologists (ASA) class, prolonged anesthesia, and the necessity for intraoperative vasopressor use.

The preoperative evaluation of patients with PAH should consist of a comprehensive history and physical examination focusing on the severity and etiology of PAH, the presence of comorbidities, and the patient's functional capacity. Risk stratification is crucial for assessing potential perioperative risk and optimizing the patient's condition before surgery [25]. A multidisciplinary approach involving pulmonologists, cardiologists, and anesthesiologists is imperative for effective patient management.

Before surgery, it is vital to optimize medical therapy for PAH. Treatment with targeted therapies, such as phosphodiesterase-5 inhibitors, endothelin receptor antagonists, and prostacyclin analogs, should be optimized to achieve the best possible preoperative status [25]. Whatever the optimized PAH treatment regimen is, it should be continued in the perioperative period.

The choice of anesthesia must be carefully considered, as different anesthetic agents have varying effects on the pulmonary vasculature. Therefore, it is essential to choose agents that maintain or reduce PVR, preserve RV function, and avoid myocardial depression [1,2,25,31-35].

Intraoperative considerations

During the intraoperative management of patients with PAH [22-26], the anesthesiologists must consider comorbid conditions such as PE, dysrhythmia, infection, electrolyte imbalance, and anemia. The primary intraoperative anesthetic considerations for PAH patients include preload, SVR, rate and rhythm, and PVR. Hemodynamic objectives encompass maintaining mean arterial pressure (MAP) of ≥60 mmHg, SBP of ≥80 mmHg, systemic arterial oxygen saturation of 94%-100%, RAP of <10 mmHg, mPAP of <55 mmHg if possible, PAOP of 8-12 mmHg, and cardiac index (CI) of ≥2.2 L/minute/m^2^ [12].

Anesthesia providers should employ a lung-protective strategy using low tidal volume (6 mL/kg, plateau pressure of <30 cm H_2_O) and optimize oxygenation through increased fraction of inspired oxygen (FiO_2_) rather than positive end expiratory pressure (PEEP) during mechanical ventilation. It is also crucial to prevent RV ischemia and acute RV failure, initiating mechanical circulatory support if warranted (mechanical fluid removal via ultrafiltration, intra-aortic balloon pump {IABP}, or extracorporeal membrane oxygenation {ECMO}) [24,25].

Meticulous airway management is necessary during anesthesia induction in PAH patients to avoid increasing pulmonary pressure. Prolonged apnea can result in a dangerous elevation of arterial carbon dioxide and the reduction of arterial oxygen, leading to progressively rising PVR and the worsening of PAH. Rapid-sequence induction (RSI) and intubation are uniquely helpful for minimizing apnea time as much as possible. PAH and PH patients, when intubation and positive pressure ventilation are required due to the nature of the surgery or if an unacceptably high risk of aspiration is present, do not tolerate well the dangerous combination of decreased arterial oxygen and increased arterial carbon dioxide on PVR, especially in patients with severe PAH or PH that comes with any degree of apnea. However, RSI, in PH or PAH patients with a low risk of aspiration, should only be utilized in a patient with a proven airway because if an unanticipated difficult airway for intubation is encountered, all the benefits of RSI are lost with prolonged intubation attempts [15,22]. The patients at a high risk of aspiration on induction require RSI in order to minimize the risk of aspiration, which in a PH or PAH patient could be lethal. Invasive monitoring, such as arterial catheterization, and central venous pressure and access are indicated in severe PAH or PH and/or PAH or PH with RV dysfunction or RHF. Pulmonary artery catheters can be considered in patients with severe PAH or PH, although the potential issues of causing arrhythmias with placement must be weighed against any potential benefit. Transesophageal echocardiography should be utilized in patients with severe PAH or PH and PH or PAH with RV dysfunction or RHF for the intraoperative monitoring of RV function and observation for acute PAH crises. Invasive monitoring and access should be considered in all patients with PH or PAH with normal RV function.

Intraoperative hypotension must be avoided, as it can induce a reflex increase in SVR, leading to acute elevations in PVR and worsening PAH. Excessive fluid administration should be circumvented, as it can cause RV dysfunction. Vasoconstrictors such as phenylephrine and norepinephrine should be administered cautiously as they elevate PVR further, while vasopressin use as a vasoconstrictor is uniquely suited in maintaining MAP in PH or PAH patients given the more minimal effects on PVR versus SVR combined with a decreased likelihood for excessive tachycardia as compared to epinephrine. Positive pressure ventilation (PPV) is potentially hazardous when used in PAH patients. Lung-protective ventilation strategies involving low tidal volumes, adequate PEEP, and limiting peak inspiratory pressures should be employed to prevent the worsening of PAH and RV dysfunction [25,29].

Preserving optimal fluid balance during surgery is crucial to avoid volume overload, which can result in RV failure. Careful fluid administration guided by invasive hemodynamic monitoring and the use of diuretics when necessary is advised. Some authors propose that maintaining a slightly negative fluid balance could benefit these patients [25,29].

The selection of anesthesia agents is critical in patients with PAH, as they can significantly impact pulmonary vasculature. Therefore, agents that minimize the effect on PVR and RV functions should be utilized. Inhalational agents such as sevoflurane and isoflurane have vasodilatory effects on the pulmonary vasculature. Therefore, they are particularly beneficial when appropriately employed in PAH patients. Intravenous agents, including propofol and etomidate, can be used for induction due to their minimal effects on PVR [25,29].

Opioids should be administered judiciously, as they can cause respiratory depression, increase arterial carbon dioxide, and decrease arterial oxygen, resulting in elevated PVR and the worsening of PAH. Remifentanil, a short-acting opioid, is a highly favorable alternative for these patients. Neuromuscular blocking agents should be selected based on their cardiovascular effects. Cisatracurium and rocuronium are preferred due to their minimal impact on PVR [25,29].

Regional anesthesia (RA) should be considered on an individual basis. It can provide excellent pain relief and reduce the need for opioids. However, it also has the potential to cause significant hemodynamic changes and impair compensatory mechanisms in patients with PAH [25,29].

Hemodynamic monitoring is essential in the intraoperative management of PAH patients. Invasive arterial blood pressure monitoring is recommended. Central venous or pulmonary artery catheterization should be uniquely considered for high-risk patients or complex surgeries with significant PAH or RV dysfunction. Advanced monitoring techniques, such as transesophageal echocardiography, can help assess RV function and guide fluid and inotropic therapy [25,29,36,37].

Postoperative considerations

The postoperative management of PAH patients [22,24-26] should include vigilant intensive care unit (ICU) monitoring for prompt detection and treatment of complications such as PAH crisis, RV failure, and arrhythmias. Pain control should be optimized, and opioids should be carefully administered. They can cause respiratory depression, increasing PVR through elevated arterial carbon dioxide and decreased arterial oxygen levels. The patients should be extubated in a supine position to prevent sudden increases in PVR. Moreover, postoperative fluid management should be conservative to avoid RV dysfunction and the worsening of PAH. If given the nature of the surgery performed, patient characteristics, or perioperative circumstances, the decision is made to go to the ICU intubated; the process of extubation may need to be more prolonged and controlled so as to avoid even the moderately elevated risk of extubating too early leading to patient issues with apnea, acute respiratory failure, and/or the need for re-intubation with all the attendant issues of restarting PPV in PAH or PH patients. The patients may have their muscle relaxation reversed earlier if the conditions that led to the patient being brought to the ICU intubated have subsided or are subsiding; however, it is imperative to avoid having the ventilator dyssynchrony with the patient as the potentially high airway pressures create high intrathoracic pressures, which can worsen PH or PAH acutely.

Anesthesia providers should avoid exacerbating PVR and RV failure postoperatively, continue the patient's medications, and consider ICU admission. In addition, intraoperative hemodynamic goals should be meticulously monitored and maintained to prevent the further deterioration of the patient's condition. Postoperative considerations include avoiding the exacerbation of PVR and RV failure, monitoring hemodynamic parameters, and assessing for complications such as bleeding or infection. The patients continue to take all their PH or PAH medications as prescribed or as soon as possible if they are unable to provide those medications as prescribed (postoperative patients who are strictly not allowed any oral intake but who are prescribed oral medications for their PH or PAH).

After surgery, patients with moderate/severe or class III/IV PAH or PH and with PH or PAH with RV dysfunction or RHF should be closely monitored in the ICU. Their medications should be continued but titrated down slowly as the patient's ventilation approaches its preoperative level. A multidisciplinary approach involving the pulmonologist, cardiologist, and anesthesiologist is crucial for ensuring optimal outcomes for these high-risk patients.

Pain control is critical during the postoperative period, as pain can lead to increased sympathetic tone, tachycardia, and elevated PVR. Therefore, a multimodal approach to analgesia, including regional techniques, intravenous analgesics, and non-opioid adjuncts, should be used to minimize the risk of respiratory depression and worsening PAH [25,29].

Managing PAH patients undergoing noncardiac surgery necessitates thorough preoperative evaluation, the optimization of medical therapy, and careful intraoperative management, focusing on the choice of anesthetic agents, PPV strategies, and optimal fluid balance. In addition, postoperative care should emphasize vigilant monitoring, adequate pain control, and prompt management of complications.

PAH is a clinical condition characterized by elevated pulmonary arterial pressure, potentially leading to right heart failure, organ dysfunction, and mortality. Numerous recent studies have explored the relationship between anesthesia and patients with PAH undergoing noncardiac surgery.

Research has investigated the associations between general anesthesia (GA) and regional anesthesia (RA) in PAH patients. It has been concluded that GA and RA can be safely applied to PAH patients, ensuring vigilant monitoring and managing of hemodynamic parameters. However, these studies emphasize the need for further investigation to determine the most appropriate anesthesia technique for this patient population.

Some experts stress the importance of individualizing anesthetic management for PAH patients. They advocate for a comprehensive preoperative evaluation, including echocardiography, and a collaborative approach involving anesthesiologists, surgeons, and cardiologists. Furthermore, they emphasize the necessity of a detailed intraoperative monitoring plan, potentially encompassing continuous invasive blood pressure monitoring, central venous pressure monitoring, and transesophageal echocardiography when required.

Some researchers argue that GA may be less favorable than RA for PAH patients undergoing noncardiac surgery. They highlight the importance of maintaining hemodynamic stability and avoiding sudden blood pressure fluctuations, which can exacerbate PAH symptoms. Additionally, they indicate that GA could result in a higher likelihood of postoperative complications, such as respiratory depression and hypotension, which are detrimental to PAH patients.

These studies demonstrate that anesthesia professionals must possess expertise in managing PAH patients during surgery. This includes a thorough preoperative evaluation, an appropriate patient selection, and an extensive intraoperative monitoring plan. Moreover, anesthesiologists must be skilled in managing PAH patients' hemodynamic and mechanical aspects, as improper management could have catastrophic consequences.

When selecting an anesthesia technique, practitioners should consider the patient's needs and weigh the risks and benefits of GA and RA. Although both methods can be safely administered to PAH patients, individual factors such as the patient's comorbidities, surgical procedure, and tolerance to RA should be considered.

Intraoperative management should maintain hemodynamic stability and prevent sudden blood pressure changes. This may involve administering vasopressors and inotropes and managing fluid cautiously. Anesthesiologists should also be prepared to address potential complications, including right ventricular failure and PAH crisis.

Postoperative care for PAH patients necessitates specialized attention, encompassing respiratory support, fluid balance optimization, and pain and anxiety management. Furthermore, anesthesia professionals must collaborate with the surgical team and other medical specialists to ensure a seamless postoperative experience and minimize complications for PAH patients.

The perioperative management of PAH patients presents a complex and challenging task for anesthesia professionals. A profound understanding of pulmonary hypertension pathophysiology and expertise in the patient's hemodynamic and mechanical aspects are crucial for successful perioperative management. In addition, GA and RA techniques can be safely applied to PAH patients. Still, the choice must be individualized based on the patient's specific needs and the surgical procedure.

A collaborative approach involving the anesthesiologist, surgeon, and cardiologist is essential for optimizing patient outcomes. In addition, preoperative evaluation, intraoperative monitoring, and postoperative care should be tailored to the unique needs of PAH patients, emphasizing hemodynamic stability maintenance and complication prevention.

Anesthesia professionals must be knowledgeable in managing PAH patients to ensure optimal surgical outcomes. By staying current with the latest research and understanding the complexities of PAH patients, anesthesia professionals can play a crucial role in reducing morbidity and mortality in this challenging patient population.

Anesthesia providers must be familiar with the impact of anesthesia on PAH patients. A comprehensive understanding of the hemodynamic and mechanical aspects of the anatomy is vital to prevent morbidity and mortality. Moreover, anesthesia providers can offer valuable insights to the surgeon when assessing a patient's candidacy for surgery, ultimately helping to ensure the best possible outcome.

## Conclusions

In the context of an ever-evolving field, anesthesia professionals must remain up-to-date with the latest research and advances in managing patients with pulmonary hypertension. By comprehending the challenges and complexities associated with these patients, anesthesia providers can effectively contribute to the overall reduction of morbidity and mortality in this population.

In summary, managing patients with PAH during surgery is a complex and challenging task that requires a deep understanding of the pathophysiology and expertise in hemodynamic and mechanical aspects. Anesthesia professionals must be proficient in preoperative evaluation, intraoperative monitoring, and postoperative care for these patients, with an emphasis on maintaining hemodynamic stability and preventing complications. A collaborative approach involving the anesthesiologist, surgeon, and cardiologist is crucial for optimizing patient outcomes. By staying informed and adaptable, anesthesia professionals can significantly impact the care and outcomes of patients with PAH.
